# Small Fibre Neuropathy Is Associated With Impaired Vascular Endothelial Function in Patients With Type 2 Diabetes

**DOI:** 10.3389/fendo.2021.653277

**Published:** 2021-04-14

**Authors:** Akihiko Ando, Michiaki Miyamoto, Naoko Saito, Kazuhiko Kotani, Hideki Kamiya, Shun Ishibashi, Mitra Tavakoli

**Affiliations:** ^1^ Department of Internal Medicine, Nishio Hospital, Nishio, Japan; ^2^ Division of Endocrinology and Metabolism, Department of Internal Medicine, Jichi Medical University, Shimotsuke, Japan; ^3^ Division of Diabetes, Department of Internal Medicine, Aichi Medical University, Nagakute, Japan; ^4^ Department of Internal Medicine, Aiseikai Clinic for Internal Medicine and Gynecology, Kuki, Japan; ^5^ Division of Community and Family Medicine, Centre for Community Medicine, Jichi Medical University, Shimotsuke, Japan; ^6^ Department of Laboratory Medicine, Jichi Medical University, Shimotsuke, Japan; ^7^ Diabetes and Vascular Research Centre, NIHR Exeter Clinical Research Facility, University of Exeter Medical School, Exeter, United Kingdom

**Keywords:** diabetic polyneuropathy (DPN), small fibre neuropathy, corneal confocal microscopy, RH-PAT, endothelial dysfunction, macroangiopathy

## Abstract

Diabetic polyneuropathy (DPN) and endothelial dysfunction are prevalent complications of diabetes mellitus. Currently, there are two non-invasive markers for endothelial dysfunction: flow-mediated dilation and reactive hyperaemia peripheral arterial tonometry (RH-PAT). However, the relationship between diabetic small fibre neuropathy and macroangiopathy remains obscure thus far. Corneal confocal microscopy (CCM) has emerged as a new diagnostic modality to assess DPN, especially of small fibre. To clarify the relationship between diabetic small fibre neuropathy and vascular dysfunction, we aimed to determine the functions of peripheral nerves and blood vessels through clinical tests such as nerve conduction study, coefficient of variation in the R-R interval, CCM, and RH-PAT in 82 patients with type 2 diabetes. Forty healthy control subjects were also included to study corneal nerve parameters. Correlational and multiple linear regression analyses were performed to determine the associations between neuropathy indices and markers for vascular functions. The results revealed that patients with type 2 diabetes had significantly lower values for most variables of CCM than healthy control subjects. RH-PAT solely remained as an explanatory variable significant in multiple regression analysis for several CCM parameters and vice versa. Other vascular markers had no significant multiple regression with any CCM parameters. In conclusion, endothelial dysfunction as revealed by impaired RH-PAT was significantly associated with CCM parameters in patients with type 2 diabetes. This association may indicate that small fibre neuropathy results from impaired endothelial dysfunction in type 2 diabetes. CCM parameters may be considered surrogate markers of autonomic nerve damage, which is related to diabetic endothelial dysfunction. This study is the first to report the relationship between corneal nerve parameter as small fibre neuropathy in patients with type 2 diabetes and RH-PAT as a marker of endothelial dysfunction.

## Introduction

The pathophysiology and disease progression of diabetic polyneuropathy (DPN), which is the most frequent diabetic complication (1), remain unclarified as compared to those of other diabetic microvascular complications such as retinopathy or nephropathy. The term ‘DPN’ principally means “diabetic symmetric sensorimotor polyneuropathy” and partly includes diabetic autonomic neuropathy. Furthermore, DPN may lead to sudden death from autonomic neuropathy and to debilitating amputations. The reason why DPN has received inadequate attention, with exacerbated severity, is due to the lack of simple, objective measuring tools for the diagnosis and estimation of therapeutic effects. Nerve conduction study (NCS) is performed to assess large fibre neuropathy objectively. Small fibre neuropathy has recently attracted more attention than before because of the awareness of its importance in patients’ quality of life and mortality. Approximately 70–90% of peripheral nerves are small fibres (mostly unmyelinated). Small unmyelinated C fibres convey warm and cold thermal perception. Cutaneous terminals of C fibres respond to heat, pain, and chemical stimuli. Cold sensation and nociception are related to myelinated A-δ nerve fibres (2). Small fibre neuropathy is, by pathological definition, a neuropathy of small-diameter nerve fibre such as A-δ and C fibres. Various methods support the diagnosis of small fibre neuropathy. Physical signs such as the presence and distribution of sensory loss and pain, gait impairment, and dysautonomic symptoms and quantitative sensory testing were utilised. The gold standard for diagnosis is the estimation of intra-epidermal nerve fibre density (IENFD); however, the procedure is somewhat invasive and time-consuming. In vivo corneal confocal microscopy (CCM) is used widely as a non-invasive and simple device because all corneal nerves consist of small C and A-δ fibres. Functionally, corneal nerve fibres penetrating the corneal stroma are primarily sensory (3) and contain almost no autonomic nerves. However, several previous reports suggested that corneal nerve changes can enable the detection of diabetic autonomic neuropathy (4–6).

Our previous studies have shown that diabetes patients with small fibre neuropathy exhibit a reduction in the number of corneal nerve fibre bundles and loss of complexity, including a reduction in corneal nerve fibre density (CNFD), corneal nerve fibre length (CNFL), and corneal nerve branch density (CNBD) (7). Additionally, corneal nerve fibre width (CNFW) or corneal nerve fibre area (CNFA) changes due to small fibre neuropathy. The multinational normative values for corneal nerve morphology were proposed in 2015 (8). The 0.05th quartile values for age and gender are usable as provisional diagnostic criteria for diabetic small fibre neuropathy.

Although the aetiology of DPN, which is one of the diabetic microvascular complications and vascular factors are the mainstay of its progression, is not fully understood, both metabolic and vascular changes contribute to its development. Micro-vasculatures, called vasa vasorum, supply blood to large blood vessels, macro-vasculature, from outside of the vessels. Therefore, micro- and macrovascular damages are inseparably intertwined. Macrovascular impairments, i.e., arteriosclerosis, are reported to be associated with several DPN markers. The two complications have a significant relationship with the incidence of diabetic foot syndrome (1).

Macrovascular damage occurs in several phases: endothelial dysfunction, arterial stiffness, and the resulting structural sclerosis. Endothelial dysfunction is the earliest stage of an atherosclerotic process. Two non-invasive markers of endothelial dysfunctions, namely, flow-mediated dilation (FMD) and reactive hyperaemia peripheral arterial tonometry (RH-PAT), are currently available (9). A comparison of the results of FMD and DPN was reported previously (10, 11). RH-PAT estimates have not been assessed from the standpoint of DPN. Moreover, the relationship between diabetic small fibre neuropathy and macroangiopathy has not been evaluated adequately.

In a previous study, we reported that the cardiovascular ankle index (CAVI) has a significant relationship with the results of NCS and markers of large fibre neuropathy in patients with type 2 diabetes (12). Using part of the same cohort, in that we could examine neuropathy and vascular markers, we investigated the relationship of markers of small and large diabetic neuropathy with markers of vascular changes, especially those of endothelial dysfunction. As an assessment tool of small fibre neuropathy, CCM parameters have one substantial limitation: the cornea is avascular for transparency; hence, CCM parameters may not be dependent on vascular changes. Therefore, the relationship between corneal nerves and vascular markers may well be almost insignificant.

We investigated cross-sectionally the association of RH-PAT and other physiological vascular markers with corneal nerve parameters, which were evaluated by confocal microscopy, and other neuropathy markers such as NCS. Additionally, the relationship between CCM parameters and other neuropathy markers, such as CVR-R and Neuropathy Gradings, were examined with this population.

## Materials and Methods

### Subjects

This is a cross-sectional, retrospective, and observational study, in which 112 inpatients with type 2 diabetes at the Jichi Medical University, Japan, were recruited between April 2014 and May 2015. The sample size was determined using larger sample CCM data (standard deviation, mean) for corneal nerve fibre length (CNFL), which is the most reliable CCM parameter, in a past report (13), calculated according to the formula, n =λ^2^C^2^/e^2^, where n: sample size, λ: confidence level (1.96), C = standard deviation (calculated from standard error)/mean of larger samples, and e: allowable error rate (0.05). The required sample size for CNFL was as follows: 116.2 for no diabetic retinopathy, 124.9 for non-proliferative diabetic retinopathy, and 163.4 for proliferative diabetic retinopathy. Therefore, the determined sample size was not larger than 116.2. The registered inpatients with type 2 diabetes in this study were from the Endocrinology and Metabolism ward of Jichi Medical University Hospital. We obtained written consent from all the subjects. The following terms were the exclusion criteria: age above 75 years, those with renal failure requiring regular haemodialysis, or after chemotherapy, or with peripheral artery disease, neurodegenerative diseases (including Parkinson’s disease, multiple sclerosis, dementia), cerebrovascular disease, and entrapment neuropathy. After recruitment, 30 patients were excluded for the lack of necessary data for further analysis or infringing on the exclusion criteria, and 82 subjects were used for analysis. The institutional review board of Jichi Medical University approved this study. Diabetic retinopathy and nephropathy were staged according to the definitions in the previous report (12). For comparison of CCM data, we included 40 controls’ CCM data. Healthy controls were recruited at the Ishibashi Clinic, Japan.

### Physical Examinations and Laboratory Measurements

The procedure of physical examinations and laboratory measurements were reported previously (12). General medical assessments, such as body height, body weight, blood pressure, medical history taking, and blood sampling, were performed for every patient. Body mass index calculations were performed using the following formula: body weight (kg) divided by the square of body height (m). Blood pressure measurement was performed in the supine position twice in a quiet room using an automated sphygmomanometer, and the recorded values were the mean levels. Mean atrial blood pressure approximations were obtained using the following formula: systolic blood pressure − diastolic blood pressure/3 + diastolic blood pressure. The definition of hypertension was blood pressure above 140/90 mmHg or taking antihypertensive medication. Smoking status and the duration of diabetes were recorded as part of medical history taking. Blood sample collections were performed in the morning after 12 hours of fasting. Using high-performance liquid chromatography, we determined glycated haemoglobin (HbA1c) levels. HbA1c of the International Federation of Clinical Chemistry unit (mmol/mol) converted from the NGSP value was presented together with the National Glycohemoglobin Standardization Program (NGSP) equivalent value (%). Enzymatic assays were performed to determine fasting plasma glucose, total cholesterol (TC), high-density lipoprotein cholesterol (HDL-C), and triglyceride (TG) levels. Calculation of low-density lipoprotein cholesterol (LDL-C) levels was performed according to the Friedewald formula. Latex agglutination turbidimetry method was followed to measure cystatin C levels.

### Assessment of Diabetic Polyneuropathy

Clinical neuropathy assessment was carried out as described in a previous report (12).

We questioned every patient for their neuropathic symptoms, i.e., on somatic symptoms such as bilateral numbness, tingling, and prickling sensation, and dysesthesia of the toes and soles of both feet. Additionally, autonomic symptoms such as orthostatic hypotension, persistent constipation, and diarrhoea were checked. For neurological signs, we examined the Achilles tendon reflex. We considered this reflex as “decreased” when there was a response for both legs in the knee-standing position (reinforcement method) with a percussion hammer or as “disappeared” when there was no response, even with such a reinforcement method. We tested a vibratory perception using a 128 Hz tuning fork. The shortening of the perceptible time for a fork vibration regarded as attenuation of vibration perception was fewer than 10 seconds. We performed Semmes-Weinstein monofilament tests to identify the presence of anaesthesia. The patients lay down and closed their eyes; then monofilaments were pressed onto the top and bottom of both feet until the tips of the filaments started to bend. When patients could not perceive the touch of monofilaments that were thinner than number 3.84 after changing the diameter of the filaments to the thicker, we estimated the patients as having sensory attenuation. We assess muscle weakness and atrophy by a manual muscle test for the extensor digitorum brevis muscle in both feet.

The proposed criteria for the diagnosis of small fibre neuropathy were clinical signs (pinprick and thermal sensory loss and or allodynia and or hyperalgesia) and quantitative sensory testing and IENFD (14). In this study, instead, the presence of small fibre neuropathy was determined using a multinational normative data set of CCM parameters for each age and gender (6).

The definition of ‘probable’ DSPN in Toronto consensus is ‘the presence of a combination of symptoms and signs of neuropathy including any two or more of the following: neuropathic symptoms, decreased distal sensation, or unequivocally decreased or absent ankle reflexes’ (15). We did not assess ‘confirmed’ DSPNs in the same consensus because they require the presence of an abnormality of NCS or validated measures of small fibre neuropathy (with class 1 evidence). It was not sure whether any abnormal values in NCS, such as upper limbs, should be adopted or not. Also, we had not conducted skin biopsy, validated measures of small fibre neuropathy, and corneal confocal nerve imaging as they are not regarded as fully certified measures of small fibre neuropathy yet (15, 16).

All symptoms and signs of DPN were added up and classified into five stages according to diabetic neuropathy stages by the Diabetic Neuropathy Study Group in Japan as in the previous paper (12, 17).

As partly validated measures of small fibre neuropathy, we used the results of corneal nerve imaging by confocal microscopy. As normative values, multinational normative datasets were adopted [8].

The coefficient of variations in the R-R interval (CV_R-R_) at rest and deep breathing were measured as previously reported (12).

The NCS measurements were previously stated (12). We recorded electrophysiological grading of DPN by NCS of all patients (16, 17). Details of classification are as follows. Grade 0: With no abnormality. Grade I: tibial F-wave latency prolongation, decreased tibial MCV (< 42 m/s) or decreased sural SCV (< 42 m/s), which reflect the degeneration of myelinated nerve fibre with the maximum diameter. Furthermore, the appearance of A wave, peroneal CMAP lowering (< 2 mV), which reflect nerve degeneration localised in the most distant site. Grade II: sural SNAP lowering (2-5 µV), which reflects the decreased density of myelinated nerve fibres around the ankle. Grade III: tibial CMAP lowering (2–5 mV), which demonstrates a failure of muscle strength maintenance mechanism at the most distal site, or sural SNAP severe lowering (< 2 µV) which reflects highly decreased density of myelinated nerve fibres around the ankle. Grade IV: tibial CMAP severe lowering (< 2 mV) which presents the elimination of motor units or abolition of motor nerve fibre around foot (18, 19).

### Corneal Confocal Microscopy (CCM)

All study subjects were assessed with Corneal Module^®^ [HRT II RCM]; Heidelberg Engineering GmbH, Heidelberg, Germany).

Subjects’ each eye was anaesthetised with a drop of benoxil^®^ ophthalmic solution 0.4% (oxybuprocaine hydrochloride, Santen Japan). Then, a large drop of GenTeal^®^ lubricant eye gel (hypromellose; Novartis Switzerland) was applied to the tip of the lens before attaching the disposable cap (tomocap^®^). Finally, we applied Scopisol^®^ solution (hydroxyethylcellulose Senju Pharma Japan) to the left eye. The ocular lens with Tomocap^®^ was slowly advanced until the cornea was in contact with the cap. We used Genteal^®^ as the coupling agent between the cornea and the lens with cap. In the chosen frames, images analyses were to quantify the following parameters. Corneal nerve fibre length (CNFL) [the total length of all nerve fibres and branches (millimetre per square millimetre)], corneal nerve fibre density (CNFD) [the total number of all main fibres per square millimetre], corneal nerve branch density (CNBD) [the total number of all branching points per square millimetre], tortuosity, and tortuosity-standardised corneal nerve fibre length [CNFL/corneal nerve fibre tortuosity] (20) were used to define corneal nerve fibre damage. Additionally, we checked CNFA [the total nerve fibre area mm^2^ per mm^2^], and CNFW [The average nerve fibre width mm per mm^2^] as corneal nerve parameters (21, 22). The whole examination took approximately 5 minutes for the left eye of each subject. All images were captured using the ‘section’ mode. Five non-overlapping pictures per patient from the centre of the cornea were selected. Selected images were analysed using semiautomated image analysis software CCMetrics and fully automated image analysis software ACCMetrics (licensed by University of Manchester) (4, 23, 24).

### Measurement of RH-PAT

The subjects were asked to lie in the supine position as for FMD, and the set probes on the bilateral index fingers. Before the occlusion of the brachial artery, the baseline measurement was for 5 minutes. The occlusion of the non-dominant arm maintained for 5 minutes above 200 mmHg or at least 50 mmHg above the systolic blood pressure. After the occlusion was released, a post-occlusion measurement continued for 5 minutes (hyperaemic period). RH-PAT detected plethysmographic pressure changes in the bilateral index fingertip caused by the arterial pulse and translated this to a peripheral arterial tone (PAT) (25). The measuring device was EndoPAT2000^®^ (Nihon Kohden Corporation, Tokyo, Japan).

### Measurement of the Other Vascular Markers

These markers were measured as reported in past reports (FMD (26), CAVI (12), brachial-ankle pulse wave velocity (baPWV) (27), maximum intima-media thickness (max IMT), and plaque score (PS) (28) of carotid artery. The intra-observer coefficient of variation for the IMT measurement was 2.8% (29).

### Statistical Analysis

Statistical analyses were performed using Stata SE version 14 (StataCorp. College Station, TX, USA). Presentations of clinical profiles of recruited patients were as means ± standard deviation. To assess the statistical difference in all markers between subjects with DPN and without DPN, we made the following tests: Wilcoxon rank-sum test in markers of continuous value without normal distribution, T-test with equal variance in markers of continuous value with normal distribution and equal variance, T-test with unequal variance in markers of continuous value with normal distribution and unequal variance, and chi-square test in markers of category value.

We assessed correlations by Spearman’s rank correlation coefficient. Then, we made simple and multiple linear regression analysis to explore the determinants of neuropathy indices and arteriosclerosis markers.

We included biologically plausible predictors in the model. These predictors are as follows: age, gender, body height, presence of obesity (body mass index BMI ≧ 25.0), mean arterial pressure (MAP), presence of dyslipidaemia, HbA1c, current smoking, cystatin C. We defined dyslipidaemia as triglyceride (TG) levels > 39 mmol/L; HDL-C levels < 10 mmol/L; LDL-C levels > 36 mmol/L; or taking medications.

The variance inflation factor estimated multilinearity.

‘r’ designates coefficients (standardised) of simple regression. ‘β’ designates coefficients of multiple regression using corneal parameters as the dependent variable, and ‘β’’ uses vascular markers as the dependent variable. These abbreviations appear as r/β/β’ in **Tables 4**, **5** and **Supplementary Tables 4, 5**. In **Table 4**, β signifies coefficients (standardised) of multiple regression using neuropathy parameters as the dependent variable, and β’ means those using RH-PAT as the dependent variable. In **Table 4**, β signifies coefficients (standardised) of multiple regression using corneal parameters as the dependent variable, and β' means those using CVR-R as the dependent variable. In **Supplementary Table 4**, β signifies coefficients (standardised) of multiple regression using neuropathy stage as the dependent variable, and β’ means those using CVR-R as the dependent variable. In **Supplementary Table 5**, β signifies coefficients (standardised) of multiple regression using corneal parameters and vascular markers as the dependent variable, and β’ means those using neuropathy stage as the dependent variable. 

## Results

### Clinical Profiles of the Subjects

Most of the subjects were elderly and middle-aged, and the female/male proportion was 36.6/63.4%. The mean duration of diabetes was about 12 years. Their diabetes was poorly controlled at the time of blood sampling because their hospitalisations were for improvement of their glycaemic status. Subjects that were habitual drinkers were 56.1%, but we excluded alcohol addicts to remove the bias of alcoholic neuropathy. Percentage of hypertension and dyslipidaemia were 64.6 and 73.2%, respectively. Complicated diabetic microangiopathies other than neuropathy were retinopathy and nephropathy at 58.5% and 35.3%, respectively. Presence of probable diabetic sensorimotor neuropathy by Toronto consensus leads to a significant difference only in cystatin C (**Table 1**).

**Table 1:** Clinical profile of the study subjects.

	Total (n=82) (Except Retinopathy)	Patients without Neuropathy (n=40)	Patients with Neuropathy (n=42 (except Retinopathy))	P-value	Tests
Age (years)	56.8 ± 11.7 (31〜74)	56.8 ± 11.7 (31〜74)	56.8 ± 11.9 (32〜74)	0.93	1
Gender (female/male)	36.6/63.4 ( 30/52)	35.0/65.0 (14/65)	38.1/61.9 (16/26)	0.77	4
Body height (cm)	164.2 ± 8.9 (140.3〜181.7)	164.9 ± 9.1 (140.3〜181.7)	163.5 ± 8.8 (142.0〜181.0)	0.45	2
Body weight (kg)	74.7 ± 16.9 (35.0〜120.4)	74.0 ± 18.5 (35.0〜107.7)	76.4 ± 15.5 (48.3〜120.4)	0.36	2
Body mass index (kg/m^2^)	27.8 ± 5.5 (16.2〜51.6)	27.8 ± 5.8 (16.2〜43.9)	27.8 ± 5.2 (19.0〜51.6)	0.92	1
Duration of diabetes (years)	11.9 ± 8.2 (1〜36)	11.4 ± 9.1 (1〜36)	12.3 ± 7.4 (1〜29)	0.36	1
Family history of diabetes (%)	70.7 (58/82)	72.5 (29/40)	69.0 (29/42)	0.73	4
Fasting plasma glucose [(mmol/L (mg/dL)]	8.8 ± 2.7 (4.4〜18.3) (157.9 ± 48.1 (80〜329))	8.3 ± 2.2 (4.4〜12.9) (149.7 ± 38.8 (80〜233))	9.2 ± 3.0 (5.0〜18.3) (165.8 ± 54.8 (90〜329))	0.26	1
HbA1c [(mmo/ml (%)] (NGSP)	82.7 ± 22.4 (37.7〜160.1) (9.7 ± 2.1 (5.6〜16.8)	78.8 ± 18.1 (44.3〜121.9 9.4 ± 1.7 (6.2〜13.3))	86.8 ± 25.4 (37.7〜160.1 10.1 ± 2.3 (5.6〜16.8))	0.19	1
Glycoalbumin (%)	23.5 ± 7.2 (10.4〜48.7)	21.9 ± 5.3 (13.5〜39.0)	25.1 ± 8.3 (10.4〜48.7)	0.09	1
Systolic blood pressure (mmHg)	128.1 ± 15.3 (97.0〜177.0)	125.7 ± 14.3 (97.0〜177.0)	130.3 ± 14.3 (101.5〜166.5)	0.16	1
Diastolic blood pressure (mmHg)	80.1 ± 8.9 (64.5〜107.0)	79.9 ± 9.0 (64.5〜107.0)	80.3 ± 9.0 (65.0〜103.5)	0.42	2
Pulse pressure (mmHg)	48.0 ± 11.2 (26.5〜83.5)	45.8 ± 10.6 (27.5〜78.0)	50.0 ± 11.5 (26.5〜83.5)	0.04*	2
Mean arterial pressure (mmHg)	96.7 ± 11.9 (78.7〜152.0)	95.2 ± 9.8 (78.7〜125.0)	98.3 ± 13.5 (79.8〜152.0)	0.40	1
Use of ARB or ACEI (%)	34.1 (28/82)	25.0 (10/40)	42.9 (18/42)	0.09	4
Hypertension (above140/90 or medication) (%)	64.6 (53/82)	57.5 (23/40)	71.4 (30/42)	0.19	4
Total Cholesterol [(mmol/L (mg/dL)]	4.4 ± 1.0 (2.6〜8.1) (169.3 ± 38.7 (102〜313))	4.5 ± 1.0 (2.6〜6.7) (172.3 ± 38.1 (102〜259))	4.3 ± 1.0 (2.8〜8.1) (166.5 ± 39.5 (110〜313))	0.43	1
TG [(mmol/L (mg/dL)]	1.5 ± 0.8 (0.6〜6.1) (136.8 ± 72.3 (49〜543))	1.5 ± 0.7 (0.6〜3.7) (130.0 ± 64.6 (49〜331))	1.6 ± 0.9 (0.6〜6.1) (143.3 ± 79.3 (50〜543))	0.26	1
HDL-C [(mmol/L (mg/dL)]	1.2 ± 0.3 (0.7〜2.7) (45.9 ± 13.2 (25〜106))	1.2 ± 0.4 (0.8〜2.7) (45.7 ± 13.7 (30〜106))	1.2 ± 0.3 (0.6〜2.3) (46.1 ± 12.8 (25〜90))	0.71	1
LDL-C [(mmol/L (mg/dL)] (Friedewald)	2.5 ± 0.9 (1.1〜4.6) (98.6 ± 34.1 (43〜177))	2.6 ± 0.9 (1.1〜4.6) (100.9 ± 34.5 (43〜177))	2.4 ± 0.8 (1.2〜4.5) (91.0 ± 32.0 (45〜175))	0.19	1
non HDL-C [(mmol/L (mg/dL)]	3.2±1.1 (1.7〜4.6) (122.5 ± 39.1 (65〜284))	3.2 ± 0.9 (1.7〜5.1) (122.5 ± 35.9 (65〜200))	3.1±1.1 (1.8〜7.3) (119.8 ± 42.2 (69〜284))	0.30	1
Use of Statin (%)	50.0 (41/82)	47.5 (19/40)	52.4 (22/42)	0.66	4
Dyslipdaemia (%) (TG>3.9 HDL<1.0 LDL>3.6 or medication)	73.2 (60/82)	75.0 (30/40)	71.4 (30/42)	0.72	4
Retinopathy (%) (SDR onwards) (n=81)	59.3 (48/81)NDR 34 SDR 19 PPDR 10 PDR 18	45.0 (18/40)NDR 22 SDR 9 PPDR 4 PDR 5	70.7 (29/41)NDR 12 SDR 10 PPDR 6 PDR 13	0.07	4
Nephropathy (%) (microalbuminuria onwards)	35.3 (29/82)1: 53 2: 15 3:12 4:2	30.0 (12/40)1: 28 2: 8 3:3 4:1	40.4 (17/42)1: 26 2: 7 3:9 4:1	0.36	4
Creatinine [(μmol/L (mg/dL)	2.0 ± 37.1 (28.3〜238.7) (0.93 ± 0.42 (0.32〜2.70))	81.6 ± 43.9 (38.9〜238.7) (0.9 ± 0.5 (0.4〜2.7))	82.4 ± 29.8 (28.3〜192.7) (0.9 ± 0.3 (0.3〜2.2))	0.25	1
CystatinC (mg/dL)	1.0 ± 0.4 (0.6〜2.7)	1.0 ± 0.4 (0.6〜2.7)	1.1 ± 0.3 (0.7〜1.9)	<0.01*	1
Smoking (%) (Current Smoker only)	31.7 (26/82) Ex-smoker 28.0 (23/82)	57.5 (23/40) Ex-smoker 27.5 (11/40)	61.9 (26/42) Ex-smoker 28.6 (12/42)	0.75	4
Alcohol (%) (excluding chance drinker)	22.0 (18/82)	30.0 (12/40)	14.3 (6/42)	0.10	4
Habitual exercise (%)	39.0 (26/82)	45.0 (18/40)	33.3 (14/42)	0.28	4
Family history of ischaemic heart disease (%)	11.0 (9/82)	15.0 (6/40)	7.1 (3/42)	0.26	4
Family history of cerebrovascular disease (%)	26.8 (22/82)	32.5 (13/40)	21.4 (9/42)	0.26	4

Tests performed to assess the significant difference in all markers between subjects with DPN and those without DPN are Wilcoxon rank-sum test, Student t-test with equal variance, Student t-test with unequal variance, and chi-square test. ARB, angiotensin II receptor blocker; ACEI, angiotensin-converting enzyme inhibitor; TG, triglyceride; HDL-C, high-density lipoprotein cholesterol; LDL-C, low-density lipoprotein cholesterol; NDR, no diabetic retinopathy; SDR, simple diabetic retinopathy; PPDR, preproliferative diabetic retinopathy; PDR, proliferative diabetic retinopathy. *There is a significant statistical difference between those with DPN and those without DPN.

### Profiles of Neuropathy and Arteriosclerosis Markers of the Subjects

**Table 2** is presenting these results. The percentage of probable diabetic sensorimotor polyneuropathy (DSPN) by Toronto consensus and of probable diabetic small fibre neuropathy assessed by CCM was 51.2% (42/82) and 45.1% (37/82), respectively. The results of diabetic neuropathy stages by the Diabetic Neuropathy Study Group in Japan and of electrophysiological grading of DPN by NCS suggested that the subjects varied remarkably in neuropathy severity. The following neuropathy or vascular markers discriminated those with probable diabetic sensorimotor neuropathy by Toronto consensus and those without: tortuosity coefficient, CNFL/Tortuosity coefficient, CV_R-R_ deep breathing, neuropathy stage by Diabetic Neuropathy Study Group in Japan, and electrophysiological grading of DPN by NCS. This neuropathy criterion distinguished tibial MCV, Peroneal CMAP, Sural SNAP, Median F-wave distal latency, Median F-wave conduction velocity, and neuropathic symptom and signs (except autonomic symptoms) too. On the other hand, there was no significant difference between patients with probable neuropathy by Toronto consensus and those without in CCM parameter other than tortuosity coefficient and CNFL/Tortuosity coefficient, presence of probable diabetic small fibre neuropathy assessed by confocal corneal microscopy, and any vascular markers (**Supplementary Table 1**).

**Table 2:** Profiles of neuropathy and endothelial dysfunction markers of the subjects.

	Total (n=82) (Except Retinopathy)	Patients without Neuropathy (n=40)	Patients with Neuropathy (n=42 [except Retinopathy)]	P-value	Tests
CNFL^a^ (mm/mm^2^)	22.7 ± 5.1 (5.9〜38.5)	23.4 ± 5.6 (5.9〜38.5)	22.1 ± 4.5 (11.6〜30.6)	0.12	2
CNFD^a^ (number/mm^2^)	27.1 ± 7.5 (7.50〜57.5)	28.1 ± 8.2 (7.5〜57.5)	26.1 ± 6.7 (15.0〜39.4)	0.21	1
CNBD^a^ (number/mm^2^)	83.9 ± 35.2 (1.2〜164.4)	87.6 ± 35.1 (1.2〜164.4)	80.3 ± 35.2 (16.2〜148.1)	0.18	2
Tortuosity coefficient	0.2 ± 0.0 (0.1〜0.3)	0.2 ± 0.0 (0.1〜0.2)	0.2 ± 0.0 (0.1〜0.3)	0.04*	2
CNFL/Tortuosity coefficient	143.8 ± 45.2 (63.0〜338.2)	152.9 ± 45.3 (86.7〜338.2)	135.2 ± 43.8 (63.0〜262.8)	0.04*	1
CNFL^b^ (mm/mm^2^)	14.9 ± 3.1 (3.9〜22.9)	15.0 ± 3.1 (3.9〜22.9)	14.7 ± 3.1 (8.2〜22.1)	0.72	1
CNFD^b^ (number/mm^2^)	23.4 ± 6.6 (5.0〜43.7)	23.7 ± 6.6 (5.0〜43.7)	23.2 ± 6.8 (10.0〜35.0)	0.57	1
CNBD^b^ (number/mm^2^)	39.7 ± 16.4 (0.0〜90.0)	40.1 ± 13.5 (0.0〜62.5)	39.4 ± 18.8 (3.7〜90.0)	0.42	3
CNFA^b^ (μm^2^/mm^2^)	4945.4 ± 1408.5 (1780.0〜10280.0)	4898.5 ± 1276.2 (1780.0〜838.0)	4990.0 ± 1538.2 (2760.0〜10280.0)	0.67	1
CNFW^b^ (μm/mm^2^)	207.8 ± 10.6 (188.2〜248.0)	209.5 ± 11.0 (188.2〜248.0)	206.2 ± 10.0 (188.4〜226.2)	0.21	1
CVR-R resting (%) (n=77)	2.3 ± 1.4 (0.4〜6.8)	2.5 ± 1.2 (0.9〜6.8)	2.2 ± 1.4 (0.4〜5.7)	0.09	1
CVR-R deep breathing (%) (n=77)	4.9 ± 2.9 (0.5〜13.4)	5.8 ± 2.7 (2.4〜12.1)	4.1 ± 2.9 (0.5〜13.4)	<0.01**	1
Neuropathy stage by Diabetic Neuropathy Study Group in Japan (%)	I: 50.0, II: 9.8, III: 7.3, IV: 23.2, V: 9.8	I: 95.0, II: 2.5, III: 0.0, IV: 0.0, V: 2.5	I: 7.1, II: 16.7, III: 14.3, IV: 45.2, V: 16.7	<0.01**	4
Electrophysiological grading of diabetic polyneuropathy by nerve conduction study (%)	0: 13.4, I: 11.0, II: 36.6, III: 34.2,IV: 4.9	0: 15.0, I: 10.0, II: 52.5, III: 20.0, IV: 2.5	0: 9.5, I: 14.2, II: 21.4, III: 47.6, IV: 7.1	0.02*	4
Presence of probable diabetic sensorimotor neuropathy in Toronto consensus (%)	51.2 (42/82)				
Presence of probable diabetic small fibre neuropathy assessed by confocal corneal microscopy (%)	45.1 (37/82)	40.0 (16/40)	50.0 (21/42)	0.36	4
RH-PAT	1.7 ± 0.5 (1.0〜3.3)	1.7 ± 0.4 (1.0〜2.9)	1.7 ± 0.5 (1.1〜3.3)	0.78	1

Data are presented as mean± standard deviation; ^a^Image analysis using CCMetrics; ^b^Image analysis using ACCMetrics. We present the data of the proportion of subjects out of institutional normative values after the mean and standard deviation of each nerve conduction parameter of the subjects. Tests performed to assess the significant difference in all markers between subjects with DPN and those without DPN are the same as those shown in **Table 1**.

CNFL, corneal nerve fibre length; CNFD, corneal nerve fibre density; CNBD, corneal nerve branch density; CVR-R, coefficient of variation in electrocardiogram R-R interval; RH-PAT reactive hyperaemia peripheral arterial tonometry.

*p<0.05, **p<0.01.

### Comparison of CCM Markers Between the Controls and Subjects

**Figure 1:**
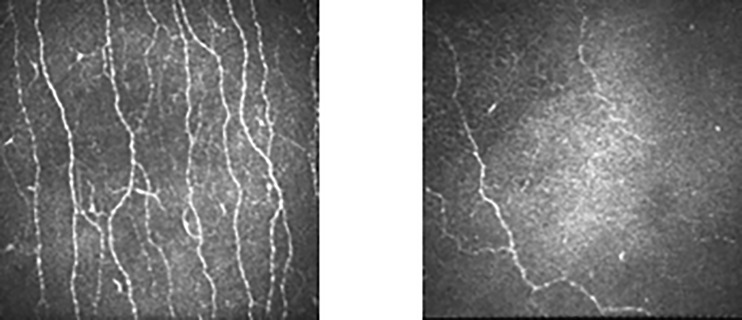
Comparison of the corneal nerve image between control subjects and patients matched for age (61 years old) and sex (female).

Comparison of the results of CCM markers between the controls and subjects demonstrated that the results of all CCM markers of the subjects were significantly lower than those of nondiabetic controls (**Table 3**). Representative corneal images of the control and subject groups are in **Figure 1**. The subjects were relatively older, but their ages did not differ significantly from those of the controls (Data not shown). When the subjects were limited to ages below 70 years old, the results of all CCM markers were significantly lower than those of nondiabetic controls (Data not shown).

**Table 3:** Comparison of the results of CCM markers between the control subjects and patients.

	Controls	Subjects	p value
Age	56.8 ± 11.7	53.9 ± 8.1	0.09
Gender(Female: Male)	30:52	14:26	0.86
CNFL^a^(mm/mm^2^)	19.9 ± 4.0	14.9 ± 3.1	<0.01
CNFD^a^(number/mm^2^)	33.8 ± 7.6	23.4 ± 6.7	<0.01
CNBD^a^(number/mm^2^)	60.8 ± 37.5	39.7 ± 16.4	<0.01

CCM, corneal confocal microscopy; CNFL, corneal nerve fibre length; CNFD, corneal nerve fibre density; CNBD, corneal nerve branch density.

a Analyses using ACCMetrics..

### Correlation, and Simple, Multiple Linear Regression Analysis Between CCM Parameters and Markers for Endothelial Dysfunction

CCM markers had no significant correlations with RH-PAT. Simple linear regression analysis indicated that RH-PAT had significant regression with CCM parameters. They include tortuosity coefficient, CNFL/tortuosity coefficient by CCMetrics (C), CNFL, and CNBD by ACCMetrics (A). RH-PAT had no significant correlation and regression with NCS markers (**Table 4**).

**Table 4:** Correlation and simple and multiple linear regression analysis of CCM markers with vascular endothelial function markers.

	RH-PAT
CNFL^a^ (mm/mm^2^)	0.06; -0.11
CNFD^a^ (number/mm^2^)	-0.01; -0.09
CNBD^a^ (number/mm^2^)	-0.06; -0.08
Tortuosity coefficient^a^	0.19; 0.27*/0.31*/0.29*
CNFL/Tortuosity coefficient^a^	-0.16; -0.30**/-0.28*/-0.26*
CNFL^b^ (mm/mm^2^)	-0.17; -0.24*/-0.25*/-0.22*
CNFD^b^ (number/mm^2^)	-0.12; -0.20
CNBD^b^ (number/mm^2^)	-0.16; -0.22*/-0.19/-0.19
CNFA^b^ (mm^2^/mm^2^)	<0.01; -0.16
CNFW^b^ (mm/mm^2^)	0.03; 0.11
Tibial DL (msec)	0.01; 0.07
Tibial MCV(m/sec)	-0.11; -0.12
Tibial CMAP(mV)	-0.07; -0.12
TibialFWDL(msec)	0.15; 0.17
Peroneal DL (msec)	0.08; 0.15
Peroneal MCV(m/sec)	-0.12; -0.13
Peroneal CMAP(mV)	-0.19; -0.18
Sural SCV (m/sec)	-017; -018
Sural SNAP (μV)	-0.16; -0.20
Median FWDL(msec)	0.09; 0.14
Median FWCV (m/sec)	-0.09; -0.13
CV_R-R_ resting	-0.15; -0.18
CV_R-R_ deep breathing	-0.14; -0.19

Spearman’s rank-sum correlation coefficients for all the patients; standardised coefficient of simple regression r and of multiple regression β, β’ (see text) for all the patients. About the other explanatory variables, see text. *p < 0.05, **p < 0.01. ^a^Image analysis using CCMetrics ^b^using ACCMetrics CCM: corneal confocal microscopy; CNFL, corneal nerve fibre length; CNFD, corneal nerve fibre density; CNBD, corneal nerve branch density; CVR-R, coefficient of variation in electrocardiogram R-R interval; DL, distal latency; MCV, motor nerve conduction velocity; CMAP, compound motor action potential; FWDL, F-wave distal latency; SCV, sensory nerve conduction velocity; SNAP, sensory nerve action potential; FWCV, F-wave conduction velocity; RH-PAT, reactive hyperaemia peripheral arterial tonometry.

CAVI had a significant simple regression with CNFL by C and with CNBD by C and A. PWV had a significant simple regression with CNBD by C and A.

CAVI, PWV, FMD, max IMT, and PS had significant correlations with and significant simple regressions with several CCM parameters but did not remain as an explanatory variable significant in multiple linear regression analysis for any CCM markers. Whereas, CAVI and PWV, arterial stiffness markers, and max IMT and plaque score had significant simple and multiple regression with several NCS markers. Correlation by Spearman’s rank correlation coefficients had the same trends as regression analysis (data not shown).

No correlation but significant regression between RH-PAT and CCM parameters signifies that there is only a weak relationship between the two indexes (**Supplementary Figure 1**). Still, some causal relationship, which represented as a primary and linear equation, existed. The degree of freedom adjusted coefficient of determination was small; therefore, the fitness of the data to the regression line was weak.

Contrary to expected, multiple linear regression analysis with CCM markers adjusted by arteriosclerosis risk factors (age. gender. body height, presence of obesity, mean arterial pressure, presence of dyslipidaemia, HbA1c, current smoking, cystatin C) for several CCM parameters indicated that RH-PAT remained as the only significant explanatory variable. Significant CCM parameters were tortuosity by C, CNFL/tortuosity by C, and CNFL by A. CNFL by A remained a significant explanatory variable after mean atrial pressure (MAP) in multiple regression analysis for RH-PAT, and RH-PAT remained as the sole explanatory variable significant in the multiple regression analysis for CNFL by A (**Supplementary Table 2**).

Among our subjects, vascular elasticity markers: CAVI and PWV and structural sclerosis markers: max IMT and PS had significant correlation and simple regression with each other. Endothelial dysfunction markers: FMD and RH-PAT had small Spearman rank correlation coefficient and had no significant simple regression with each other within this studied population with diabetes (**Supplementary Table 3**).

### Correlations, Simple and Multiple Regressions Between CCM Parameters and Other Markers of DPN, Such as CV_R-R_ and NCS

Almost all CCM parameters had significant simple and multiple regression results with CV_R-R_ both in resting condition and deep breathing (**Table 5**). Limited numbers of CCM parameters had significant correlations and simple, multiple regressions with some of NCS markers (Data not shown). Neuropathy stages defined by clinical symptoms and signs, and electrophysiological neuropathy grading by NCS were both significantly related to the results of CV_R-R_ (**Supplementary Table 3**). CCM had significant statistical relations with electrophysiological grading, but had no significant statistical relations with clinical neuropathy staging, except for CNFL/tortuosity coefficient (**Supplementary Table 4**). Similarly, RH-PAT had significant statistical relations with electrophysiological grading but had no significant statistical relations with clinical neuropathy staging. CAVI had significant correlations (not with clinical neuropathy staging) and no significant regression with electrophysiological neuropathy grading. The other vascular markers had no significant statistical relations with neuropathy staging (clinical and electrophysiological) (data not shown).

**Table 5:** Correlation and simple and multiple linear regression between CCM parameters and CV_R-R_.

	CV_R-R_ resting	CV_R-R_ deep breathing
CNFL^a^ (mm/mm^2^)	0.41**; 0.40**/0.40**/0.34**	0.36**; 0.35**/0.31**/0.32**
CNFD^a^ (number/mm^2^)	0.31**; 0.36**/0.35**/0.29**	0.34**; 0.34**/0.30*/0.31*
CNBD^a^ (number/mm^2^)	0.35 **; 0.34**/0.31*/0.27*	0.26*; 0.25*/0.21/0.22
Tortuosity coefficient^a^	-0.14; -0.21	-0.16; -0.23*/-0.20/-0.21
CNFL/Tortuosity coefficient^a^	0.46**; 0.45**/0.47**/0.39**	0.42**; 0.41**/0.37**/0.38**
CNFL^b^ (mm/mm^2^)	0.32**; 0.35**/0.39**/0.31**	0.32**; 0.32**/0.31*/0.31*
CNFD^b^ (number/mm^2^)	0.28*; 0.33**/0.35**/0.28**	0.34**; 0.37**/0.34**/0.34**
CNBD^b^ (number/mm^2^)	0.25*; 0.22	0.23*; 0.18
CNFA^b^ (mm^2^/mm^2^)	0.07; 0.04	0.03; -0.04
CNFW^b^ (mm/mm^2^)	-0.17; -0.15	-0.22; -0.20

Spearman’s rank-sum correlation coefficients for all the patients; Coefficients (standardised) of simple regression r, and multiple regression β, β’ for all the patients. β: independent variables were CVR-R, β’: independent variables were CCM markers. About the other explanatory variables, see text. ^a^Image analysis using CCMetrics ^b^using ACCMetrics *p < 0.05, **p < 0.01.

CNFL, corneal nerve fibre length; CNFD, corneal nerve fibre density; CNBD, corneal nerve branch density.

## Discussion

The present multiple linear regression analysis results indicated that only RH-PAT had a significant relation with CCM markers among physiological markers of arteriosclerosis.

The cornea is avascular, and the corneal nerves are nourished mainly by the aqueous humour *via* diffusion and partly by the axonal flow, which comes from the nerve cell body located in the brainstem. Corneal nerves are the offshoot of the trigeminal nerve. They originate from the principal sensory nucleus in the pons and medulla oblongata which is perfused by superior cerebellar artery and nuclear artery (a branch of the basilar artery). Therefore, corneal nerves are not likely to have direct ischaemic damage caused by macrovascular impairment, especially arterial stiffness and structural sclerosis. Probably for that reason, arterial stiffness marker, CAVI and PWV, and marker of structural sclerosis: max IMT and PS, may have no significant relation with CCM parameters (data not shown).

In this study, we adopted two sets of data for CCM parameters: CNFL, CNFD, CNBD, tortuosity coefficient by CCMetrics and CNFL, CNFD, CNBD by ACCMetrics. The former method involved image analyses using semiautomated software, which can calculate CCM parameters based on the nerve fibres traced by touch pens. On the other hand, the latter can detect nerve fibres and calculate CCM parameters automatically. The procedure by CCMetrics is time-consuming but can detect all the nerve fibres, which may be overlooked in the analyses using ACCMetrics. Also, CCMetrics can calculate the tortuosity coefficient. The standardisation of CNFL is possible using the tortuosity coefficient (20).

Endothelial dysfunction is a crucial initiator of macro- and microvascular damage and regarded as the earliest change in arteriosclerosis progression. Pathological conditions reduced the activities of the vasculature-protective, endothelial enzyme nitric oxide synthase, produced under many stimulants (for example acetylcholine) and mechanical factor (i.e., shear stress). The interferences of the endothelium-derived vasculature-contracting substances (e.g. thromboxane A2) and reactive oxygen species caused these reductions (30). One can assess endothelial dysfunctions by the actions of vasodilators secreted from the endothelial cells after the temporal occlusion of the blood flow. FMD and RH-PAT are widely available for their assessment. These two methods can detect endothelial dysfunction equally but differ in several points. FMD measures vasodilation responses after avascularisation of brachial artery, while RH-PAT estimates those of the fingertip arterial bed.

The previous report conjectured that different regions for the evaluation of endothelial function between the large conduit artery and the resistant vessels in patients might lead to different correlation results with various risk factors. FMD had correlations with traditional risk factors and RH-PAT with metabolic risk factors such as diabetes or obesity (30). Besides, the FMD measurement principle involves the reactive, one-dimensional dilation of arterial diameter, while that of RH-PAT utilises the finger plethysmogram, i.e., three-dimensional measurement of changes in volume within the fingertip. For RH-PAT sympathetic nerve influences are cancelled by bilateral measurement, while for FMD, cancellation of the influences is not possible. Nevertheless, this cancellation effect of RH-PAT may not be fully adequate, as explained below. These differences in measurement method and site may lead to inconsistent results examining the statistical relation between RH-PAT and FMD. There was a significant correlation between these two endothelial function markers in subjects with coronary artery disease (31, 32), but a weak or no association in the general population (33, 34) or subjects with hypertension (35). Autonomic, especially sympathetic activation assessed by heart rate variability, induced by 5-min forearm clamping, affects more significantly RH-PAT, rather than FMD despite the cancellation by bilateral measurement for RH-PAT (36). These differences may explain the different results of the regression analysis with CCM parameters between RH-PAT and FMD.

The plausible explanation for the significant statistical relationship between CCM parameters and RH-PAT results may be as follows: CCM parameters have significant statistical relations with CV_R-R_, a marker of cardiac autonomic functions as exemplified in Table 9. Although RH-PAT itself had no significant relationship with CV_R-R_, it had a relatively closer relationship with CVR-R among vascular markers (**Table 4**). These results may signify that CCM could pick up autonomic nerve change reflected in RH-PAT results as with CV_R-R_. The past studies which evaluated the diagnostic utility of CCM for autonomic diabetic neuropathy endorse this conjecture (4–6). RH-PAT may detect autonomic nerve stimulation related to vascular change despite the cancellation by bilateral measurements. Among many vascular markers, RH-PAT is the sole marker which has a statistically significant relation with electrophysiological neuropathy grading in multiple regression (**Supplementary Table 5**). RH-PAT may reflect more sensitively diabetic neuropathic change compared to the other vascular markers. CCM parameters are a morphometric change of corneal nerve fibres. The reason why these structural abnormalities can detect autonomic functional change apparent in RH-PAT needs further clarifications.

CAVI and PWV, arterial stiffness markers, and max IMT and plaque score, structural sclerosis markers had a significant simple and multiple regression with several NCS parameters (large fibre neuropathy parameters). However, there are no significant multiple regressions between these advanced arteriosclerosis markers and CCM parameters (small fibre neuropathy parameters). These results suggest that diabetic small fibre neuropathy may have relations with a different stage of arteriosclerosis progressions, compared to diabetic large fibre neuropathy.

This study is novel in that this is the first study examining the statistical relationship between corneal nerve parameter as small fibre neuropathy in patients with type 2 diabetes and RH-PAT as markers of endothelial dysfunction together with the other vascular markers. The results of this study may shed some light on the aetiology of DPN and pathophysiology of DPN progression.

This study has several limitations. Firstly, as it was a cross-sectional, observational study with a limited number of subjects, a sequential causal association in time-series between CCM parameters and RH-PAT cannot be assumed. To elucidate the pathophysiologic relationship, we need a more robust interventional study with large sample size. Second, we did not include background clinical data of control subjects for evaluation such as neuropathy parameters except for CCM and vascular markers and metabolic profiles, as the clinic in Japan, different from the study subjects’ institution, provided control data. Third, small fibre diabetic neuropathy was assessed in the analyses using the results of a few confocal microscopic images of corneal nerve plexus. A few selected 2-dimensional images for analyses were only a part of the 3-dimensional whole corneal nerve plexus. Analyses of 3-dimensional, whole corneal nerve plexus, considerate of the depth of the focal plane, were necessary for the precise objective evaluation of the small neuropathy in diabetes (37, 38). Fourth, the staging and classification (clinical and electrophysiological) of DPN were for Japanese patients. Therefore, there may be some ethnic bias. Furthermore, and finally, the gold standard of diabetic small fibre neuropathy is still IENFD, but we did not measure the IENFD of the studied subjects.

In summary, in this paper for the first time, we tried to establish the relation between DPN progression and worsening of arteriosclerosis severity. Parameters of small fibre neuropathy, CCM, remained the only significant explanatory variable for RH-PAT among various physiological vascular markers in multiple linear regression analysis; whereas, the results of NCS, markers of large fibre neuropathy, but not CCM, had a significant relationship with other vascular markers of arterial stiffness and structural sclerosis. The results of this study suggest that there may be a possibility that small and large fibre diabetic neuropathy had a different relationship with each stage of macroangiopathy, but further study examining the relationship between IENFD and various stages of microangiopathies are requisite in prospective clinical settings or animal experiments. CCM parameters may be a surrogate marker of autonomic nerve damage which is related to diabetic endothelial dysfunction assessed by RH-PAT.

## Data Availability Statement

The raw data supporting the conclusions of this article will be made available by the authors, without undue reservation.

## Ethics Statement 

The studies involving human participants were reviewed and approved by Jichi Medical University. The patients/participants provided their written informed consent to participate in this study.

## Author Contributions

AA was involved in the design of the study, collection and assembly of data, and drafting the article. MM and NS contributed to the collection and assembly of data and critical revision of the manuscript. KK advised on the statistical analyses of data. HK and SI performed critical revision of the manuscript for relevant intellectual content. MT contributed to the critical review of the manuscript for relevant intellectual content as well as final approval of the article. All authors contributed to the article and approved the submitted version.

## Conflict of Interest

The authors declare that the research was conducted in the absence of any commercial or financial relationships that could be construed as a potential conflict of interest.

## Abbreviations

ARB, angiotensin II receptor blocker; ACEI, angiotensin-converting enzyme inhibitor; baPWV, brachial-ankle pulse wave velocity; BMI, body mass index; CAVI, cardio-ankle vascular index; CCM: corneal confocal microscopy; CMAP, compound motor action potential; CNBD, corneal nerve branch density; CNFA, corneal nerve fibre area; CNFD, corneal nerve fibre density; CNFL, corneal nerve fibre length; CNFW, corneal nerve fibre width; CVR-R, coefficient of variation in electrocardiogram R-R, interval; DL, distal latency; DPN, diabetic polyneuropathy; DSPN, diabetic sensorimotor polyneuropathy; FMD, flow-mediated dilation; FWCV, F-wave conduction velocity; FWDL, F-wave distal latency; HDL-C, high-density lipoprotein cholesterol; IENFD, intraepidermal nerve fibre density; LDL-C, low-density lipoprotein cholesterol; MAP, mean arterial pressure; maxIMT, maximum intima-media thickness; MCV, motor nerve conduction velocity; NCS, nerve conduction study; NDR, no diabetic retinopathy; NGSP, National Glycohaemoglobin Standardization Program; PDR, proliferative diabetic retinopathy; PPDR, preproliferative diabetic retinopathy; PS, plaque score; RH-PAT, reactive hyperaemia peripheral arterial tonometry; SDR, simple diabetic retinopathy; SCV, sensory nerve conduction velocity; SNAP, sensory nerve action potential; TC, total cholesterol; TG, triglyceride.
