# Supplementary material for: Small Fibre Neuropathy Is Associated With Impaired Vascular Endothelial Function in Patients With Type 2 Diabetes
Source: Front Endocrinol (Lausanne). 2021 Apr 14;12:653277. doi: 10.3389/fendo.2021.653277 (PMC8079951; doi:10.3389/fendo.2021.653277)
Supplement: Supplementary file 1 [file DataSheet_1.docx]

**Supplementary Figure 1**: Scatter plot and linear fitted value of the relationship between RH-PAT and CNFL/tortuosity.

**CNFL/Tortuosity Coefficient**

**RH-PAT**


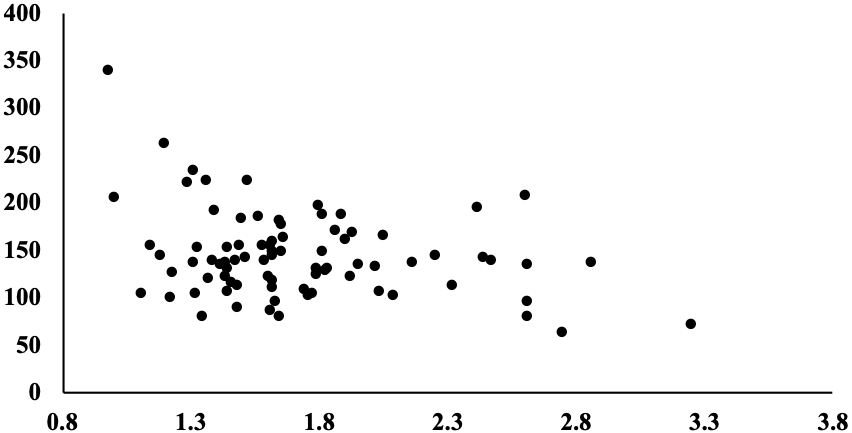


Degree of freedom-adjusted coefficient of determination: 0.0772

Regression line: CNFL/tortuosity= −29.9＊RH-PAT + 195.4

RH-PAT reactive hyperaemia peripheral arterial tonometry; CNFL corneal nerve fibre length

**Supplementary Table 1:** Profiles of neuropathy (NCS and clinical symptoms) and arteriosclerosis except RH-PAT markers of the subjects (mean± standard deviation).

|  | Total (n=82)(Except Retinopathy) | Patients without Neuropathy (n=40) | Patients with Neuropathy (n=42 (except Retinopathy)) | P-value | Tests |
| --- | --- | --- | --- | --- | --- |
| Tibial DL(ms) (<5.1) | 4.3±0.8(2.8〜7.3); 13.4(11/82) | 4.2±0.7(3.4〜6.4); 5.0(2/40) | 4.4±0.9(2.8〜7.3); 5.0(9/42) | 0.20 | 1 |
| Tibial MCV (m/s) (48.4± 5.8) | 39.2±4.5(29.2〜48.7); 75.6(62/82) | 40.7±4.1(31.8〜47.6); 67.5(27/40) | 37.8±4.5(29.2〜48.7); 83.3(35/42) | <0.01** | 2 |
| TibialCMAP (mV) (10.1<) | 9.8±4.7(0.9〜21.1); 48.7(40/82) | 11.0±3.6(1.9〜18.0); 48.7(40/40) | 8.6±5.4(1.0〜21.1); 48.7(40/42) | 0.01* | 3 |
| TibialFWDL(n=78) (ms) (44.7±6.0) | 52.8±5.2(40.4〜67.7); 64.1(50/78) | 51.5±4.9(40.4〜65.6); 64.1(21/39) | 54.2±5.2(44.1〜67.7); 64.1(29/39) | 0.02* | 1 |
| Peroneal DL(n=70) (ms) (<4.7) | 4.2±0.9(2.4〜6.5); 42.9(18/70) | 4.1±0.9(2.9〜6.5); 22.5(9/40) | 4.2±0.9(2.4〜6.4); 30.0(9/30) | 0.46 | 1 |
| Peroneal MCV (m/s)(n=70) (49.8±6.4) | 41.2±4.4(30.6〜49.9); 71.9(50/82) | 41.8±4.0(30.6〜47.6); 65.0(26/40) | 40.3±4.8(31.7〜49.9); 80.0(24/30) | 0.08 | 2 |
| Peroneal CMAP (mV) (1.9<) | 2.8±2.4(0.0〜8.5) ); 39.0(32/82) | 3.5±2.4(0.1〜8.5) ); 22.5(9/40) | 2.1±2.2(0.0〜7.4) ); 54.8(23/42) | <0.01** | 2 |
| Sural SCV(n=68) (m/sec)(46.9±6.8) | 41.8±5.1(29.7〜53.0); 26.4(18/68) | 42.1±4.7(32.0〜52.6); 23.1(9/39) | 41.5±5.7(29.7〜53.0); 23.1(9/39) | 0.33 | 2 |
| Sural SNAP (μV)(>3.3) | 3.1±2.7(0.0〜11.7) ); 63.4(52/82) | 3.5±2.2(0.0〜8.8) ); 62.5(25/40) | 2.7±3.1(0.0〜11.7) ); 64.3(27/42) | 0.04* | 1 |
| MedianFWDL(n=78) (ms) (25.5±3.6) | 28.7±3.0(21.5〜40.5); 47.4(37/78) | 27.9±2.2(21.5〜32.4); 47.4(11/38) | 29.4±3.5(22.8〜40.5); 47.4(26/40) | 0.03* | 1 |
| MedianFWCV(n=78) (m/s) (69.1±7.6) | 61.0±5.9(46.5〜73.2); 48.7(38/78) | 62.3±5.4(49.8〜72.3); 48.7(15/38) | 59.8±6.2(46.5〜73.2); 48.7(23/40) | 0.03* | 2 |
| Neuropathic symptom and signs (Rate of abnormality) |  |  |  |  |  |
| Bilateral foot symptoms (%) | 37.8 (31/82) | 10.0 (4/40) | 64.2 (27/42) | <0.01** | 4 |
| Achiles tendon reflex (%) | 39.0 (33/82) | 12.5(5/40) | 66.7 (28/42) | <0.01** | 4 |
| Vibration perception (%) | 52.4 (43/82) | 30.0(12/40) | 73.8 (31/42) | <0.01** | 4 |
| Monofilaments (%) | 42.7 (35/82) | 20.0(8/40) | 64.2 (27/42) | <0.01** | 4 |
| Autonomic symptoms (%) | 57.3 (47/82) | 50.0(20/40) | 64.2 (27/42) | 0.19 | 4 |
| Atrophy and muscle weakness of Extensor digitorum brevis (%) | 11.0 (9/82) | 2.5 (1/40) | 19.0(8/42) | 0.02* | 4 |
| FMD (n=81) | 1.7±1.2(0.2〜5.8) | 1.8±1.3(0.2〜5.5)(n=40) | 1.6±1.2(0.2〜5.8) (n=41) | 0.39 | 1 |
| CAVI (n=81) | 8.4±1.2(4.8〜11.0) | 8.6±1.3(5.9〜11.0)(n=40) | 8.2±1.1(4.8〜10.1)(n=41) | 0.09 | 2 |
| baPWV(m/s) | 1510.6±289.4(899〜2496) | 1502.2±313.8(899.0〜2496.0) | 1518.5±267.7(992.5〜2363.0) | 0.65 | 1 |
| MaxIMT (mm) (n=81) | 1.5±0.7(0.7〜3.7) | 1.5±0.7(0.8〜3.7)(n=39) | 1.5±0.6(0.7〜2.8)(n=42) | 0.86 | 1 |
| PS(n=79) | 4.5±4.0(0.0〜15.7) | 4.0±3.9(0.0〜15.7)(n=39) | 5.1±4.0(0.0〜14.8)(n=40) | 0.16 | 1 |

Data are presented as shown in Table 2.

DL distal latency; MCV motor nerve conduction velocity; CMAP compound motor action potential; FWDL F-wave distal latency; SCV sensory nerve conduction velocity; SNAP sensory nerve action potential; FWCV F-wave conduction velocity; FMD flow-mediated dilation; CAVI cardio-ankle vascular index; baPWV brachial-ankle pulse wave velocity; maxIMT maximum intima-media thickness; PS plaque score

**Supplementary Table 2:** Standardised coefficient of multiple linear regression (β, β') of all explanatory variables.

| RH-PAT | β |
| --- | --- |
| Tortuosity coefficient^a^ ; CNFL / tortuosity coefficient^a^ ; CNFL^b^ | -0.29* ;-0.26*；-0.23* |
| Age | 0.05 ; 0.05；0.06 |
| Gender | -0.08 ; -0.09；-0.94 |
| Body Height | 0.08 ; 0.08 ; 0.94 |
| Obesity | -0.26*; -0.22 ; -0.18 |
| Mean atrial pressure | 0.23* ; 0.22 ; 0.26* |
| Dyslipidaemia | -0.07 ; -0.08 ; -0.96 |
| HbA1c | -0.16 ; -0.16 ; -0.14 |
| Current Smoking | -0.03 ; <0.01 ; 0.01 |
| Cystatin C | 0.05 ; 0.05 ; 0.10 |
| Tortuosity coefficient^a^ ; CNFL / tortuosity coefficient^a^ ; CNFL^b^ | β’ |
| RH-PAT | -0.31 ; -0.28 ; -0.25* |
| Age | 0.06 ; -0.07 ; -0.04 |
| Gender | -0.04 ; -0.01 ; -0.02 |
| Body Height | -0.02 ; >-0.01 ; 0.08 |
| Obesity | 0.15 ; 0.01 ; 0.14 |
| Mean atrial pressure | -0.04 ; > -0.01 ; 0.16 |
| Dyslipidaemia | -0.01 ; -0.03 ; -0.11 |
| HbA1c | 0.13 ; -0.13 ; -0.05 |
| Current Smoking | 0.16 ; -0.03 ; >-0.01 |
| Cystatin C | 0.19 ; 0.12 ; -0.04 |

β: independent variables were RH-PAT, ∗p < 0.05, β': independent variables were CNFL a, Tortuosity coefficient b, and CNFL divided by tortuosity coefficient b. ∗ p < 0.05

RH-PAT: reactive hyperaemia peripheral arterial tonometry; CNFL: corneal nerve fibre length;

a: an image analysis using CCMetrics; b: an image analysis using ACCMetrics

**Supplementary Table 3:** Spearman's rank-sum correlation and simple linear regression between vascular markers.

|  | Correlation | Simple regression |
| --- | --- | --- |
| Endothelial dysfunction( RH-PAT:FMD) | 0.24* | 0.13 |
| Arterial stiffness(CAVI:PWV) | 0.69** | 0.55** |
| Structural sclerosis(maxIMT: Plaque score) | 0.78** | 0.80** |

*p < 0.05, **p < 0.01

**Supplementary Table 4.** Correlation and simple and multiple linear regression between CCM parameters and neuropathy stage (clinical and electrophysiological).

|  | CV_R-R_ resting | CV_R-R_ deep breathing |
| --- | --- | --- |
| Neuropathy stage (by Diabetic Neuropathy Study Group in Japan) | -0.36** ; -0.34**/-0.25*/-0.30* | -0.39** ; -0.34*/-0.31*/-0.30* |
| Electrophysiological grading of diabetic polyneuropathy by nerve conduction study | -0.25* ; -0.24*/-0.18/-0.19 | -0.32** ; -0.28*/-0.18/-0.21 |

Spearman's rank-sum correlation coefficients for all the patients. Coefficients (standardised) of simple regression, r, and of multiple regression β, β' (see text) for all the patients. β: independent variables were neuropathy stage (clinical and electrophysiological), β': independent variables were CCM parameters or RH-PAT. About the other explanatory variables, see text.

a Image analysis by CCMetrics b by ACCMetrics *p < 0.05, **p < 0.01

**Supplementary Table 5.** Correlation and simple and multiple linear regression between CCM parameters and neuropathy stage (clinical and electrophysiological).

|  | Neuropathy stage (by Diabetic Neuropathy Study Group in Japan) | Electrophysiological grading of diabetic polyneuropathy by nerve conduction study |
| --- | --- | --- |
| CNFL^a^ (mm/mm^2^) | -0.19 ; -0.19 | -0.29** ; -0.30**/-0.26*/-0.27* |
| CNFD^a^ (number/mm^2^) | -0.15 ; -0.14 | -0.28** ; -0.27*/-0.23*/-0.23* |
| CNBD^a^ (number/mm^2^) | -0.15 ; -0.17 | -0.16 ; -0.23*/-0.17/-0.17 |
| Tortuosity coefficient^a^ | 0.14 ; 0.16 | 0.34** ; 0.30**/0.31**/0.31** |
| CNFL/Tortuosity coefficient^a^ | -0.26* ; -0.24*/-0.23*/-0.23* | -0.34** ; -0.32**/-0.28*/-0.29*E87 |
| CNFL^b^ (mm/mm^2^) | -0.16 ; -0.16 | -0.31** ; -0.26*/-0.23*/-0.24* |
| CNFD^b^ (number/mm^2^) | -0.15 ; -0.16 | -0.32** ; -0.31**/-0.28*/-0.29* |
| CNBD^b^ (number/mm^2^) | -0.19 ; -0.12 | -0.16 ; -0.15 |
| CNFA^b^ (mm^2^/mm^2^) | -0.08 ; <0.01 | 0.05 ; 0.09 |
| CNFW^b^ (mm/mm^2^) | -0.02 ; <0.01 | 0.20 ; 0.21 |
| RH-PAT | 0.08 ; 0.09 | 0.22* ; 0.27*/0.26*/0.24* |
| FMD | -0.06; -0.04 | -0.06 ; -0.05 |
| CAVI | -0.06; -0.04 | 0.23* ; 0.19 |
| PWV | 0.03 ; -0.01 | 0.17 ; 0.13 |
| maxIMT | 0.07 ; 0.01 | -0.02 ; 0.01 |
| Plaque Score | 0.20 ; 0.16 | 0.09 ; 0.05 |

Spearman's rank-sum correlation coefficients for all the patients; Coefficients (standardised) of simple regression r and of multiple regression β, β' (see text) for all the patients. β: independent variables were neuropathy stage (clinical and electrophysiological), β': independent variables were CCM parameters or RH-PAT. About the other explanatory variables, see text.
